# First Evidence of *Phylloscopus collybita abietinus* in Sicily: A Morphological and Molecular Perspective

**DOI:** 10.3390/ani16010112

**Published:** 2025-12-31

**Authors:** Gea Manganaro, Renzo Ientile, Marco Mancuso, Giada Santa Calogero, Venera Ferrito, Anna Maria Pappalardo

**Affiliations:** Department of Biological, Geological and Environmental Sciences, Section of Animal Biology “M. La Greca”, University of Catania, Via Androne 81, 95124 Catania, Italy; gea.manganaro@phd.unict.it (G.M.); marco.mancuso@unict.it (M.M.); giada.calogero@phd.unict.it (G.S.C.); vferrito@unict.it (V.F.)

**Keywords:** Chiffchaff, *Phylloscopus collybita*, subspecies, morphometry, genetics, phenology, migration, Sicily

## Abstract

The “Chiffchaff complex” is a group of species with several similar subspecies, whose post-breeding distribution remains poorly understood, particularly in southern Europe. This study combines morphological and molecular approaches to investigate the subspecific composition and phenological patterns of Chiffchaffs captured in eastern Sicily. A total of 380 individuals were biometrically measured, with particular focus on wing length of P8 feather, and 81 individuals were genetically analyzed using *ND2* mitochondrial marker. Morphological analysis highlighted significant variation in P8 length between phenological groups. Assuming that a turnover of individuals from different origins may occur in the study area, genetic investigation was deemed necessary to further investigate this high morphological diversity. Phylogenetic analysis revealed high intraspecific genetic diversity and identified two subspecies in the study area: *P. c. collybita* (73%) and *P. c. abietinus* (27%). These findings genetically confirm for the first time the presence of *P. c. abietinus* in Sicily and suggest a complex pattern of seasonal co-occurrence between populations. This work contributes to the understanding of Chiffchaff migration ecology and underlines the importance of integrating ringing data with molecular tools in Mediterranean biodiversity hotspots.

## Simple Summary

The Common Chiffchaff *Phylloscopus collybita* is a small migratory songbird widely distributed across Europe and Asia. Within this species, several subspecies exist that are often indistinguishable in the field. This makes it difficult to understand where different populations migrate or spend the winter. In this study, we combined morphological and genetic analysis to investigate the Chiffchaff populations occurring in eastern Sicily—a key migratory stopover and wintering site in the central Mediterranean. Over a period of nearly ten years, we collected biometric data from 380 individuals and obtained samples for genetic analyses from a subset of 81 birds. A first clue of the diversity between Chiffchaffs came from the statistical analysis of wing length measurements. Genetic analysis revealed the presence of two subspecies: *P. c. collybita* from central and western Europe, and *P. c. abietinus* from northern and eastern Europe. This is the first genetic confirmation of *P. c. abietinus* wintering in Italy. Our findings suggest that eastern Sicily serves as a convergence zone for multiple Chiffchaff populations, highlighting its importance for the conservation of migratory birds. This study also shows how combining morphology and genetics can improve our understanding of migration patterns and diversity in cryptic bird species.

## 1. Introduction

The “Chiffchaff complex” includes a broad group of species and subspecies, widely distributed across the Palearctic and morphologically very similar or even indistinguishable from one another [1]. For this reason, they were long considered a single species: *Phylloscopus collybita* (Vieillot, 1817). In recent decades, genetic and bioacoustic research [2,3,4,5,6] has led to the division of this former taxon into four species: *Phylloscopus ibericus* (Ticehurst, 1937) from the Iberian Peninsula, *Phylloscopus canariensis* (Hartwig, 1886) from the Canary Islands, *Phylloscopus sindianus* (Brooks, 1880) from the mountainous regions of the Caucasus and Himalayas, and *Phylloscopus collybita*, which occupies the rest of Europe and Asia. In particular, this latter species includes six subspecies: *P. c. collybita*, *P. c. abietinus*, *P. c. tristis*, *P. c. brevirostris*, *P. c. caucasicus*, and *P. c. menzbieri* [7,8]. Among these, the only subspecies exhibiting sufficiently distinct morphological traits to allow for identification by visual inspection alone is *P. c. tristis* [9,10].

The European breeding range of the Chiffchaff is mainly occupied by the nominal subspecies *P. c. collybita* in western and central Europe, *P. c. abietinus* in northern and northeastern Europe, and *P. c. tristis* in Siberia and central Asia, with overlapping areas where individuals exhibit intermediate morphological and behavioral characteristics [5]. As a migratory species, European populations redistribute between Europe and Africa during the winter, undertaking long migrations as far as the Sahel region in sub-Saharan Africa [9]. Although individuals belonging to populations that come from different parts of the breeding range may encounter each other in wintering areas or during migratory stopovers, these events do not result in gene flow between the populations [11]. Thus, despite the extensive movements, the species’ fidelity to breeding sites over long periods has led to isolation, resulting in intraspecific and interspecific genetic diversity. In this regard, the phylogeographic study conducted by Raković et al. [12] clarified the delineation of the breeding ranges of Chiffchaff complex subspecies. However, their post-breeding distribution remains generally understudied, with research carried out in the Netherlands [13,14], Germany [15], Ireland [16], Great Britain [17,18], and the United Arab Emirates [19].

However, due to the difficulty in distinguishing the various subspecies in the field, knowledge of their distribution during the non-breeding season remains poorly understood or entirely unknown for some areas. For these reasons, aspects concerning geographic distribution, taxonomy, and phenology of species belonging to the Chiffchaff complex have been genetically investigated, mainly through the use of some mitochondrial markers: *ND2* [12], *CytB* [16,17,18], *CytB* combined with *ND1*, *ATP8/ATP6*, and *ND3/tRNA-ARG* [15,20]. Considering that Sicily represents both a wintering area and a fundamental flyway crossroad for migrating birds between Europe and Africa, the present study aims to apply for the first time morphological and molecular analysis to bird ringing in this region. The purposes are to investigate the intraspecific variability between Chiffchaff complex and to better comprehend the origins of individuals wintering in the study area or migrating through it. Therefore, this study represents a contribution to the understanding of important ecological aspects of Chiffchaff, a generally understudied species in Italy.

## 2. Materials and Methods

### 2.1. Study Area and Bird Ringing Methodology

The intraspecific variability of Chiffchaff was investigated in an eastern Sicilian area belonging to the Integral Nature Reserve “Complesso Immacolatelle e Micio Conti” (37°33′31″ N 15°06′56″ E). The study area is situated at an elevation of 200 to 300 m above sea level in the foothills of Mount Etna, in proximity to the city of Catania. The local habitats have been shaped by longstanding agro-pastoral practices, resulting in a heterogeneous vegetation mosaic. The arboreal layer is primarily composed of *Quercus virgiliana* (Ten.) and *Quercus amplifolia* Gus. (*Q. pubescens* group), with *Celtis australis* L. also present. Scattered individuals of *Olea europaea* L., remnants of historical cultivation, are also found. A rich Mediterranean maquis is also present at the site, characterized by the dominance of *Euphorbia dendroides* L., in association with various sclerophyllous species including *Olea oleaster* (Hoffmg. et Lk.), *Pistacia terebinthus* L., and *Rhamnus alaternus* L. The herbaceous layer is characterized by species typical of dry uncultivated environments, such as *Hyparrhenia hirta* (L.), *Carlina corybosa* L., and *Dittrichia graveolens* (L.).

The sampling material was collected as part of a monitoring project about passerines bird species. This project is conducted in a constant effort ringing station adhering to the “MonITRing” project, coordinated by the National Ringing Center (CNI-ISPRA), based in Bologna, which represents the Italian node of the EURING project.

The period of the study goes from 2016 to 2024. During these nine years, ringing sessions were carried out three times per month, with each session scheduled to fall within a different ten-day period of the month. In this way, each month can be divided in three periods: from the 1st to the 10th day, from the 11th to the 20th day, and from the 21st to the 30th day. A short label was assigned to each period for each month under analysis (for example, DEC I, DEC II, and DEC III refer, respectively, to the first, second, and third period of December). Sessions were also spaced at least five days apart from one another. The monitoring was suspended only between February 2020 and December 2021 because of the COVID-19 pandemic emergency.

### 2.2. Morphology and Statistical Analysis

The morphological variability was evaluated comparing wing length between groups of individuals divided in relation to their phenology.

The length of the eighth primary (commonly referred to as P8, counting from the inner to the external part of the wing, following the substitution order of feathers during the molt) was chosen as wing measure for the biometric comparison. In order to obtain this measure, we used a ruler with a needle fixed at zero and inserted at the base between P8 and P9 feathers [21].

The value of this measurement is expressed in millimeters (mm) and rounded up to a half millimeter. All the measurements discussed in this study were made from the same bird ringer (RI). Moreover, all measurement techniques carried out during bird ringing are standardized and therefore replicable from the same person or anyone else [22]. Photographical material of Chiffchaffs was also collected during the bird ringing.

Furthermore, regarding the sex of the individuals, as they were not sampled during the reproductive period, it was not possible to make a distinction on a morphological basis, nor was molecular sexing of the individuals carried out.

The entire group of studied individuals was divided in relation to their phenology. In particular, to make this division as precise as possible, the group was divided not referring generally by the month of the capture but more specifically by the ten-day periods in which captures were made (see Section 2.1).

The statistical test one-way ANOVA was applied to these groups of measurements to verify if there were statistically significant differences. A Tukey test was then conducted to compare each pair of groups. The tests were carried out with R studio, version 2024.04.2+764 [23]. For the statistical analysis, only seven groups of ten-days periods were compared, for which a dataset of measurements greater than 30 units was available. Recoveries of birds captured in more than one ten-day period were included in each one, but recoveries of birds captured in the same ten-day period were excluded.

All graphs and figures were created and edited using R studio, Excel, and Inkscape, version 1.4 (e7c3feb1, 2024-10-09).

### 2.3. Sampling and Molecular Analysis

Feather samples were collected in addition to morphological measurements, from January 2024 to December 2024, during the ringing sessions of the MonITRing project. A feather sample from each captured Chiffchaff was taken. In particular, it was chosen between the central rectrices or the inner secondaries. Samples were collected in 2 mL tubes containing a 70% alcohol solution and stored at 4 °C.

Molecular analysis was conducted starting from the DNA extraction from calamus [24]. A NucleoSpin^®^ extraction kit (Macherey-Nagel, Düren, Germany) was used following a standard extraction protocol from animal tissues. PCR (Polymerase Chain Reaction) was carried out, with a starting cycle at 95 °C for 3 min, followed by 35 cycles at 95 °C for 45 s, 57 °C for 45 s, 72 °C for one minute, and a further elongation cycle at 72 °C for 10 min. Specific primers for the amplification of ND2 mitochondrial sequence of the Chiffchaff were chosen: L5219Met (Forward) and H6313Trp (Reverse) [12,25,26]. All obtained amplicons were sequenced at Eurofins Genomics, and sequences were submitted to the NCBI GenBank database. The obtained sequences were analyzed together with other sequences downloaded from GenBank [27]. Only sequences for which the geographical origin and subspecies were specified were downloaded. The dataset was constructed using the sequences obtained in the present study, together with 5 sequences for each one of the 9 taxa of the Chiffchaff complex (*Phylloscopus ibericus*, *Phylloscopus canariensis*, *Phylloscopus sindianus sindianus*, *Phylloscopus sindianus lorenzii*, *Phylloscopus collybita tristis*, *Phylloscopus collybita caucasicus*, *Phylloscopus collybita menzbieri*, *Phylloscopus collybita abietinus*, *Phylloscopus collybita collybita*) downloaded from GenBank. Including the 3 sequences used as outgroup, belonging to the Willow Warbler *Phylloscopus trochilus*, a complete dataset of 129 sequences was analyzed.

Haplotypes were inferred from the complete sequence dataset of using the ‘haplotypes’ package [28] in RStudio version 2025.09.2+418 [23]. MAFFT version 7.490 [29] was used to carry out the alignment of the obtained haplotypes. The alignment was subsequently visualized and modified through Aliview version 1.28 [30]. To carry out the phylogenetic inference analysis, the dataset was first inspected in JModelTest2 v2.1.10 [31] in order to identify the best model of sequence evolution according to the Bayesian Information Criterion (BIC). The Hasegawa–Kishino–Yano two-parameter model (HKY) with gamma-distributed rate variation among sites (Γ) was identified by JModelTest2 as the most suitable model. The phylogeny was then constructed in BEAST 2.7.7 [32] under the Bayesian Inference criterion. The Markov Chain Monte Carlo (MCMC) was configured to run for 100 million generations, with tree sampling performed every 5000 generations. To improve exploration of the tree space, the analysis was repeated twice using different random seed points. A total of 20,000 trees were sampled per analysis, resulting in 40,000 sampled trees overall. The resulting “.log” and “.trees” files from both runs were combined using LogCombiner package to enhance the resolution. Convergence was assessed in Tracer v1.7.2 [32], where it was confirmed that the effective sample sizes (ESSs) for all parameters exceeded 200 and that all samples had been drawn from a stationary, unimodal distribution. Final tree annotation was performed in TreeAnnotator [33] using all 40,000 sampled trees and applying a 10% burn-in. Post-burn-in samples were summarized under the Maximum Clade Credibility (MCC) tree option in TreeAnnotator 2.7.7 [32]. The 95% highest posterior density (HPD) intervals and posterior clade probabilities (PP) were calculated for each node.

## 3. Results

### 3.1. Morphometric Analysis

During the study period, 220 days of bird ringing were conducted, and on 89 of these days (40.4%), the Common Chiffchaff was captured and ringed. The species was recorded between the last ten days of September and the first ten days of April, with a single isolated capture on 5 May 2017. However, the highest frequencies were recorded between the last ten days of November and the last ten days of January. The phenology of the species in the study area indicates its presence during the wintering period, pre-breeding migration, and post-breeding migration; it is not a breeding species in the study area.

The biometric measurements under analysis regarded 411 individuals, recorded 492 times. Those measurements were divided into seven groups of ten-day periods in relation to the phenology. No birds previously ringed at other ringing stations were captured. All the recoveries refer to individuals captured in the study area.

**Figure 1:**
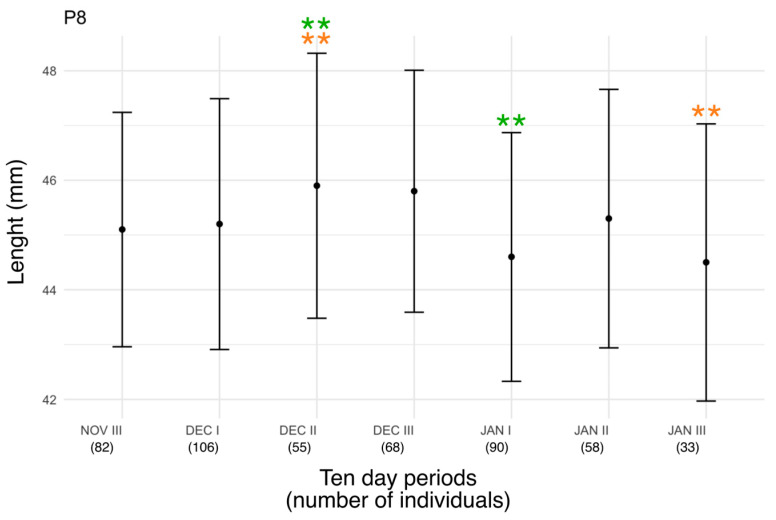
Mean length of P8 feather ± standard deviation in relation to phenology of Chiffchaff. Statistically significant differences between the groups are denoted using ** (green for the group JAN I—DEC II and orange for the group JAN III—DEC II).

A one-way ANOVA test was applied to the dataset of measurements revealed highly statistically significant differences between groups (F (6, 485) = 3.97, *p* = 0.0007), corresponding to a significance level of *** (*p* < 0.001) (Figure 1). The Tukey test revealed that two pairs of groups showed the largest and statistically significant difference level of ** (*p* < 0.01). In particular, these two pairs are JAN I—DEC II and JAN III—DEC II.

### 3.2. Molecular Analysis

Molecular analysis yielded 81 sequences of 1017 base pairs (bp) of the mitochondrial marker *ND2*, obtained from samples collected in the study area.

**Figure 2:**
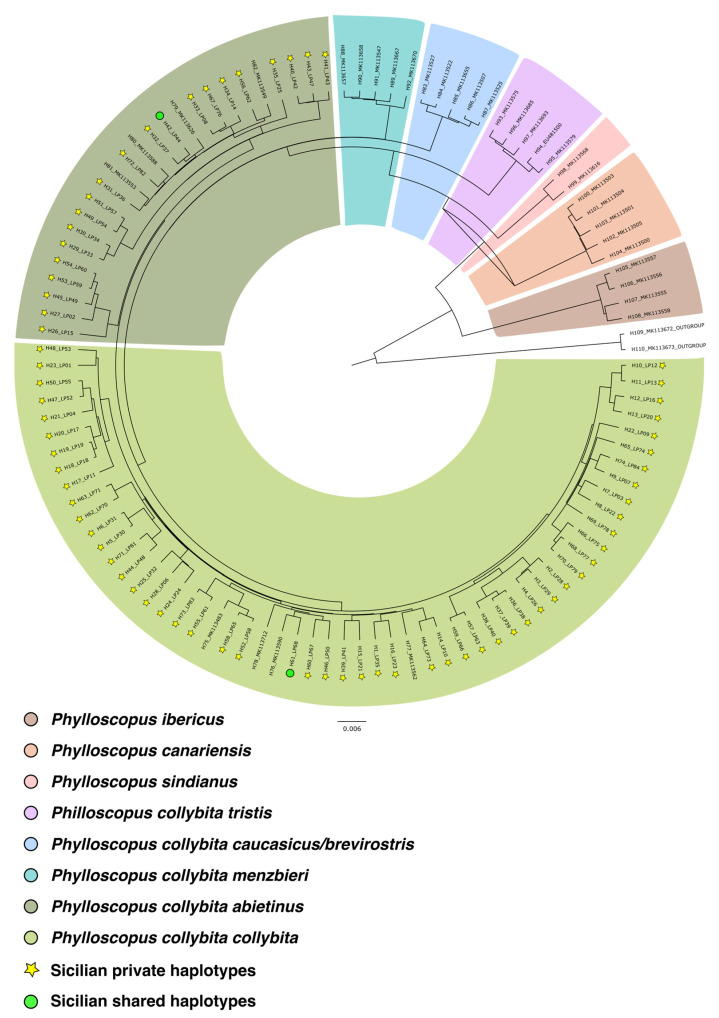
Phylogenetic tree. Eight mtDNA clades can be distinguished and are highlighted by different colored sectors. The haplotypes of individuals sampled in Sicily as part of this study are labeled as follows: ‘H’ followed by the haplotype number, an underscore, and ‘LP’ followed by the individual number. Haplotypes from sequences downloaded from GenBank are labeled with their original accession numbers from the database.

The haplotype designation resulted in the identification of 108 different haplotypes. Bayesian analysis yielded a single phylogenetic tree (Figure 2), showing the distribution of 108 haplotypes grouped into eight mitochondrial DNA clades (the two subspecies of *P. sindianus* were grouped together and graphically represented as a single sector of the tree due to their lower number of haplotypes, with only one haplotype for subspecies). Each clade corresponds to a different taxon of the complex and, according to the literature, is associated with a specific portion of the species’ breeding range. In particular, the following clades are distinguished: *Phylloscopus ibericus*, *Phylloscopus canariensis*, *Phylloscopus sindianus*, *Phylloscopus collybita tristis*, *Phylloscopus collybita caucasicus*, *Phylloscopus collybita menzbieri*, *Phylloscopus collybita abietinus*, and *Phylloscopus collybita collybita*.

Of these 108 haplotypes, 74 were found among the 81 individuals sampled in Sicily. All the sequences are deposited in GenBank, with accession number from PX512574 to PX512647 (Supplementary Table S1). Two of these 74 haplotypes are shared with individuals from different parts of the species’ breeding range. In particular, H42 (belonging to the subspecies *P. c. abietinus*) is shared by two Sicilian individuals (LP44 and LP72) and one southern Serbian individual (sequence accession number: MK113465); H61 (belonging to the subspecies *P. c. collybita*) is shared by three Sicilian individuals (LP68, LP69, and LP85) and one individual from southern Serbia (sequence accession number: MK113466). All the other haplotypes were private.

**Figure 3:**
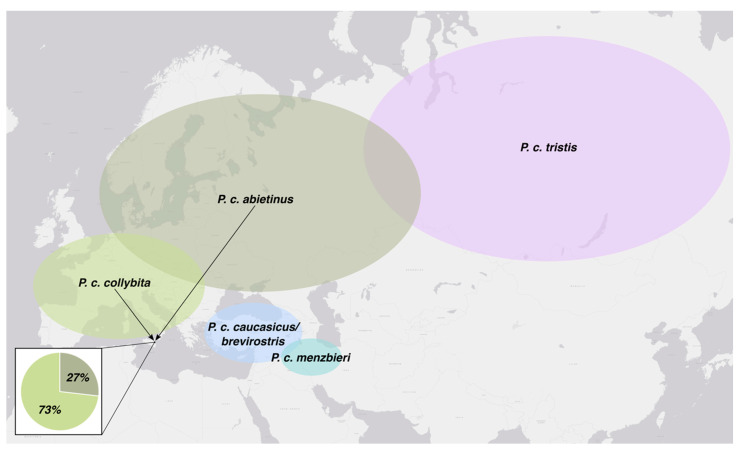
Indicative map of the breeding ranges of *Phylloscopus collybita* subspecies with frequency of two genetically confirmed subspecies (*P. c. collybita* and *P. c. abietinus*) found in the study area during wintering and migrating seasons.

**Figure 4:**
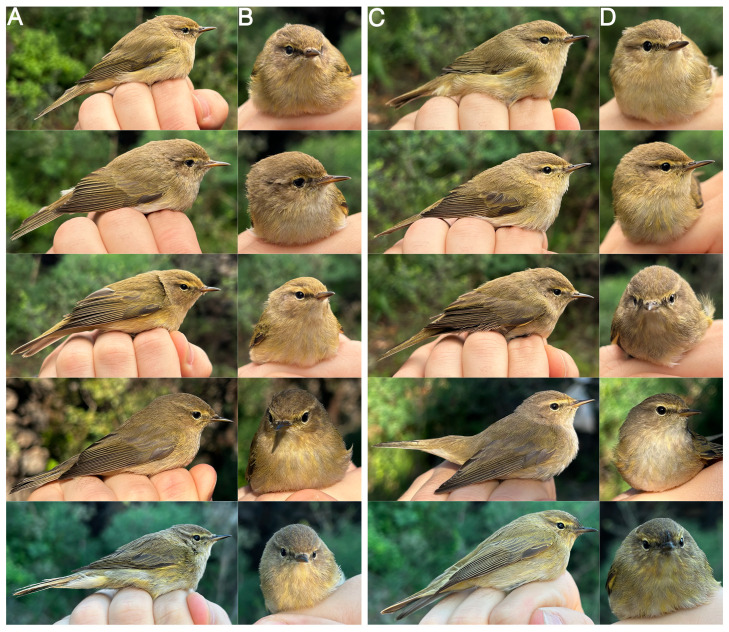
Photographic comparison between *P. c. abietinus* (**A**,**B**) and *P. c. collybita* (**C**,**D**). (**A**) Lateral view of 5 *P. c. abietinus* individuals and (**B**) frontal view of the same individuals. (**C**) Lateral view of 5 *P. c. collybita* individuals and (**D**) frontal view of the same individuals. Photos taken by Gea Manganaro.

Based on the phylogenetic analysis, the 81 Sicilian individuals belong to two of the eight clades. Specifically, 73% belong to the subspecies *P. c. collybita* and 27% to *P. c. abietinus* (Figure 3). Although phenotypically indistinguishable (Figure 4), the individuals of the two subspecies form two closely related but clearly distinct mitochondrial DNA clades.

### 3.3. Phenology

**Figure 5:**
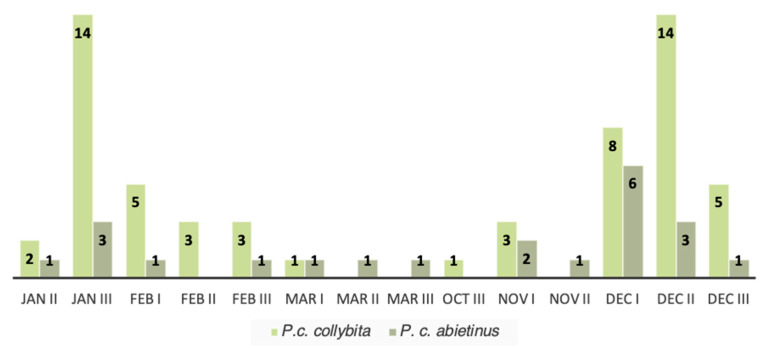
Patterns of the occurrence and the abundance of the two subspecies (*P. c. collybita* and *P. c. abietinus*) genetically identified in the study area. This graph relates in details the occurrence of the subspecies to the ten-day periods in which individuals were captured. Only time periods of 2024 in which at least one individual was captured are shown.

Based on the molecular identification of the subspecies, a histogram was constructed to easily visualize the phenological pattern of the 81 individuals during 2024. The histogram shows the pattern of the occurrence and the abundance of the two subspecies in each ten-day period in which individuals were captured (Figure 5). Ten-day periods with no birds captured were excluded. This phenological analyses of the two subspecies shows that the relative abundance of *P. c. abietinus* compared to *P. c. collybita* increases during the migration months (March and November) and decreases during the wintering months (December, January, and February).

## 4. Discussion

The results obtained in this study have provided new data on the migration ecology of the Chiffchaff species complex, which has been little studied in Italy. In detail, the study revealed the following:

- A preliminary morphological analysis revealed highly significant statistical differences in the wing length of P8 feather among individuals grouped according to their phenology, indicating a high level of intraspecific diversity in this trait;
- Molecular analysis, conducted on a subset of individuals, revealed high genetic variability. In particular, two distinct subspecies (*P. c. collybita* and *P. c. abietinus*) phenotypically indistinguishable were identified;
- As a result of the molecular findings, the phenological pattern of occurrence of the two subspecies within the study area has been determined.

Regarding the morphometric analysis, the highly statistically significant difference in P8 length detected between groups through the one-way ANOVA test suggests that birds ringed in different periods could belong to distinct populations originating from different regions of the breeding range. However, this result requires further investigation through the analysis of a larger sample size. However, currently there is insufficient evidence to rule out the possibility that the intraspecific variability observed in the P8 length is influenced by factors such as uneven representation of age classes or sexes, which are known to differ in terms of size, rather than by belonging to different populations or subspecies. In fact, *P. c. collybita* and *P. c. abietinus* exhibit a slight sexual size dimorphism (typically 0.5–1.5 mm difference) in wing length, with males generally exhibiting slightly longer wings than females. This difference is not considered to be sufficiently reliable for a certain discrimination between the two sexes, but it could be sufficient to influence the result of the statistical analysis. This latent variability may contribute to part of the observed differences in P8 length among phenological groups. Future studies including molecular sexing or larger biometric datasets could help disentangle sex-related variation from subspecific morphological differences.

Molecular analyses revealed considerable intraspecific variability at the genetic level. In fact, 74 distinct haplotypes were identified among the 81 individuals examined. Furthermore, phylogenetic analyses confirmed the presence of two distinct subspecies. Thanks to the extensive knowledge available in the literature regarding the distribution of European Chiffchaff populations, it was possible to identify the locally occurring subspecies and, indirectly, provide insights into the geographical origins of the captured individuals. For the first time in Italy and Sicily, the presence of the subspecies *P. c. abietinus* has been genetically confirmed, and its occurrence within the study area has been quantitatively assessed, with a frequency of 27%. The breeding range of *P. c. abietinus* extends across northern and eastern Europe, from the Scandinavian Peninsula and the Balkan region to the Ural Mountains in European Russia [12]. This result is of particularly important because the winter presence in Sicily of an eastern population of *Phylloscopus collybita* is not documented in the “Italian Migration Atlas” [34], which analyzes over 60,400 ringing records collected between 1982 and 2003. The atlas only reports few recoveries in Sicily of individuals originally ringed in central Europe, corresponding to the range of the subspecies *P. c. collybita*. Moreover, regarding the recoveries abroad of birds ringed in Italy, there is a total absence of data about Sicily. The “Sicilian Atlas of Biodiversity” also lacks information concerning Chiffchaffs originating from eastern Europe, suggesting central Europe as the probable origin of the individuals arriving in Sicily in autumn [35]. The checklist of the Bird of Sicily [36] assumes that both *P. c. collybita* and *P. c. abieitnus* occur in the region; however, due to the inability to distinguish the two subspecies on morphological or phenotypic characteristics, the presence of *P. c. abietinus* had never been confirmed.

Furthermore, it should be noted that unlike the findings of the present study in Sicily, in other European regions where similar studies have been conducted, the frequency of the subspecies *P. c. abietinus* is considerably lower. In particular, referring to northern Europe, the frequency of *P. c. abietinus*, during the autumn and the winter, is about 5% in the Netherlands [14] and about 6% in Great Britain and Ireland [18]. In Sweden, where both *P. c. collybita* and *P. c. abietinus* occur, ringing recoveries suggest that the former has a western migration component, whereas the latter seems to take a more eastern route [37]. Evidence of differences in migratory routes among populations belonging to the same species has also been observed in another passerine species, using stable hydrogen isotopes (δ^2^H) combined with ringing recoveries to trace the breeding areas of origin [38].

Once the subspecies identity of the 81 individuals was genetically confirmed, further investigations about their phenology were carried out. In particular, a slight increase in the relative abundance of *P. c. abietinus* compared to *P. c. collybita* was observed during the migration months. Conversely, during the wintering months, a marked increase in the relative abundance of *P. c. collybita* compared to *P. c. abietinus* was detected.

In this context, examining P8 length on a larger sample size than that analyzed in this study could reveal, in the future, a correlation between eighth-primary feather length measurements and the identified subspecies on a molecular basis. Various authors report size differences between the two subspecies [9,21,39]; however, according to van der Spek and de Knijff [13], the wing measurements alone are not a decisive feature to identify *P. c. abietinus*. Thus, the topic remains to be explored in these two Chiffchaff subspecies. However, there is already evidence within the genus *Phylloscopus* demonstrating the usefulness of biometric measurements, particularly maximum wing chord and body mass, in distinguishing between the two sister species *Phylloscopus bonelli* and *Phylloscopus orientalis* [40].

**Figure 6:**
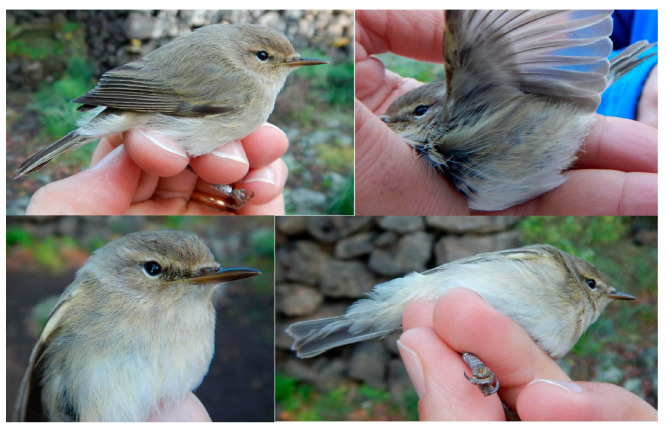
Individuals of *Phylloscopus collybita* ringed on 27 January 2017, displaying a plumage attributable to the ssp. *tristis*. Photos taken by Renzo Ientile.

Finally, the Chiffchaff complex, which was studied for the first time in the examined area, deserves further investigation as it revealed the presence of the north-eastern subspecies, previously only suspected. This finding is also supported by the identification of an individual ringed on 27 January 2017 displaying typical *P. c. tristis* plumage. However, subspecies assignment was based solely on morphological characters, as no genetic analyses were performed. This subspecies breeds in the eastern part of the Palearctic region and winters in the Indian subcontinent [41]. The identified individual exhibited typical characteristics of the *tristis* plumage, including the absence of greenish tones and a white supercilium with no yellow pigmentation. Additionally, the underparts were noticeably paler and showed a stronger contrast with the upperparts compared to the other subspecies (Figure 6).

## 5. Conclusions

For the first time in Italy and Sicily, the presence of the subspecies *P. c. abietinus* has been genetically confirmed.

Genetic investigations, combined with morphological analyses, may not yield clear insights, at least at a preliminary level, as seen in the case of the Eurasian Reed Warbler (*Acrocephalus scirpaceus*) [42]. In other cases, however, such as in the Subalpine Warbler complex [43], those studies become a valuable tool to reveal cryptic species, subspecies, or “Evolutionary Significant Units” [44].

In the present study, it has been observed that the investigation of biometric traits, particularly focused on the wing length, can already provide a first clue or indication of intraspecific variability in Common Chiffchaffs, offering guidance for further genetic investigation. Subsequently, molecular analyses proved to be a fundamental tool for the identification of cryptic taxa within the ‘Chiffchaff complex’. Differences in plumage coloration appear to be completely irrelevant, except for the individual who showed marked characteristics attributable to the subspecies *P. c. tristis*.

Despite having provided interesting results, as discussed above, this study has some limitations. In particular, there is a substantial temporal gap between the period during which biometric measurements were obtained (9 years) and the single year in which samples for molecular analyses were collected. This imbalance inevitably affects the number of individuals examined from a biometric versus a genetic perspective. For these reasons, future research will aim to address these discrepancies and to analyze a more comprehensive dataset in which both types of analyses are performed for each ringed individual.

Nevertheless, this study highlighted the potential of a research field that has been largely unexplored. Moreover, expanding the study to additional sites will undoubtedly provide new insights to better define the wintering patterns of the occurrence and the migration routes of the Common Chiffchaff subspecies in the central Mediterranean region. The high level of intraspecific diversity revealed by this study supports the key role played by certain areas in the conservation of European migratory species.

Similar studies extended to widely distributed species, where the necessary conditions are met, can provide indirect information on migratory movements and wintering areas, thereby enriching the knowledge derived from bird ringing. Moreover, such investigations offer the potential to fill knowledge gaps in geographic areas where ringing effort is low or absent. At the same time, these kinds of studies can reveal intraspecific biodiversity hotspots that might otherwise go entirely unnoticed or require years of ringing activity to be detected.

Furthermore, it would be interesting to conduct future research on breeding population of the Chiffchaff in Sicily, which were not investigated in the present study, in order to determine whether they belong to the nominal subspecies or not.

## Data Availability

The molecular data of sequences are available in GenBank. All the other data presented in this study are available on request from the corresponding authors.

## Supplementary Materials

The following supporting information can be downloaded at https://www.mdpi.com/article/10.3390/ani16010112/s1. Table S1: *Phylloscopus collybita ND2* haplotypes used in this study with accession number of the sequences deposited in GenBank.

## References

[1] Svensson L. (2001). The correct name of the Iberian Chiffchaff *Phylloscopus ibericus* Ticehurst 1937, its identification and new evidence of its winter grounds. Bull. Br. Ornithol. Club.

[2] Helbig A.J., Martens J., Seibold I., Henning F., Schottler B., Wink M. (1996). Phylogeny and species limits in the Palaearctic chiffchaff *Phylloscopus collybita* complex: Mitochondrial genetic differentiation and bioacoustic evidence. IBIS Int. J. Avian Sci..

[3] Clement P., Helbig A.J., Small B. (1998). Taxonomy and identification of chiffchaffs in the Western Palearctic. Br. Birds.

[4] Marova I.M., Fedorov V.V., Shipilina D.A., Alekseev V.N. (2009). Genetic and vocal differentiation in hybrid zones of passerine birds: Siberian and European chiffchaffs (*Phylloscopus* [*collybita*] *tristis* and *Ph.* [*c.*] *abietinus*) in the southern Urals. Dokl. Biol. Sci..

[5] Shipilina D., Serbyn M., Ivanitskii V., Marova I., Backström N. (2017). Patterns of genetic, phenotypic, and acoustic variation across a chiffchaff (*Phylloscopus collybita abietinus/tristis*) hybrid zone. Ecol. Evol..

[6] Calviño Cancela M., Piña L., Martín Herrero J. (2022). Bioacoustic differentiation of calls in the chiffchaff complex. PeerJ.

[7] Vaurie C. (1959). The Birds of the Palearctic Fauna: A Systematic Reference, Order Passeriformes.

[8] Gill F., Donsker D., Rasmussen P. (2024). IOC World Bird List (v15.1). https://www.worldbirdnames.org/new/updates/.

[9] Cramp S., Brooks D.J. (1992). Handbook of the Birds of Europe, the Middle East and North Africa. The Birds of the Western Palearctic, Volume VI. Warblers.

[10] Svensson L., Mullarney K., Zetterström D., Grant P.J. (2024). Guida degli Uccelli d’Europa, Nord Africa e Vicino Oriente.

[11] Pegan T.M., Kimmitt A.A., Benz B.W., Weeks B.C., Aubry Y., Burg T.M., Hudon J., Jones A.W., Kirchman J.J., Ruegg K.C. (2025). Long distance seasonal migration to the tropics promotes genetic diversity but not gene flow in boreal birds. Nat. Ecol. Evol..

[12] Raković M., Neto J.M., Lopes R.J., Koblik E.A., Fadeev I.V., Lohman Y.V., Aghayan S.A., Boano G., Pavia M., Perlman Y. (2019). Geographic patterns of mtDNA and Z linked sequence variation in the Common Chiffchaff and the ‘chiffchaff complex’. PLoS ONE.

[13] de Knijff P., van der Spek V., Fischer J. (2012). Genetic identity of grey chiffchaffs trapped in the Netherlands in autumns of 2009–11. Dutch Bird..

[14] van der Spek V., de Knijff P. (2021). Migrating chiffchaff taxa in the Netherlands: A 10 year genetic study. Dutch Bird..

[15] van der Spek V., Dierschke J., Copete J.L., de Knijff P. (2025). First records in north west Europe of two Common Chiffchaff *Phylloscopus collybita* taxa from the Middle East and the Caucasus. Bull. Br. Ornithol. Club.

[16] O’Mahony B., Farrer D., Collinson M. (2015). Genetic identity of wintering Common Chiffchaffs *Phylloscopus collybita* trapped in County Kerry in 2015. Ir. Birds.

[17] Collinson J.M., Shannon T., Archer P., Odin N., Riddington R., Walsh P. (2013). Genetic analysis of migrant Siberian Chiffchaffs in Britain and Ireland. Br. Birds.

[18] Collinson J.M., Murcia A., Ladeira G., Dewars K., Roberts F., Shannon T. (2018). Siberian and Scandinavian Common Chiffchaffs in Britain and Ireland a genetic study. Br. Birds.

[19] Motteau V., Campbell O., Senfeld T., Shannon T.J., Collinson J.M., Lloyd S. (2022). First occurrence of menzbieri Common Chiffchaff in the United Arab Emirates. Br. Birds.

[20] de Knijff P., te Raa M. (2024). First records of *Phylloscopus collybita brevirostris* (Strickland, 1837) and *Phylloscopus collybita caucasicus* (Loskot, 1991) in north western Europe. Zenodo.

[21] Svensson L. (1992). Identification Guide to European Passerines.

[22] Spencer R. (1972). The Ringer’s Manual.

[23] R Core Team (2016). R: A Language and Environment for Statistical Computing.

[24] Taberlet P., Bouvet J. (1991). A single plucked feather as a source of DNA for bird genetic studies. Auk.

[25] Reeves A.B., Drovetski S.V., Fadeev I.V. (2008). Mitochondrial DNA data imply a stepping stone colonization of Beringia by Arctic Warbler *Phylloscopus borealis*. J. Avian Biol..

[26] Sorenson M.D., Ast J.C., Dimcheff D.E., Yuri T., Mindell D.P. (1999). Primers for a PCR based approach to mitochondrial genome sequencing in birds and other vertebrates. Mol. Phylogenet. Evol..

[27] Benson D.A., Cavanaugh M., Clark K., Karsch Mizrachi I., Lipman D.J., Ostell J., Sayers E.W. (2012). GenBank. Nucleic Acids Res..

[28] Aktas C., Aktas M.C. (2015). Package ‘Haplotypes’. http://r.meteo.uni.wroc.pl/web/packages/haplotypes/haplotypes.pdf.

[29] Katoh K., Rozewicki J., Yamada K.D. (2019). MAFFT online service: Multiple sequence alignment, interactive sequence choice and visualization. Brief. Bioinform..

[30] Larsson A. (2014). AliView: A fast and lightweight alignment viewer and editor for large datasets. Bioinformatics.

[31] Darriba D., Taboada G.L., Doallo R., Posada D. (2012). jModelTest 2: More models, new heuristics and parallel computing. Nat. Methods.

[32] Bouckaert R., Heled J., Kühnert D., Vaughan T., Wu C.H., Xie D., Suchard M.A., Rambault A., Drummond A.J. (2014). BEAST 2: A software platform for Bayesian evolutionary analysis. PLoS Comput. Biol..

[33] Heled J., Bouckaert R.R. (2013). Looking for trees in the forest: Summary tree from posterior samples. BMC Evol. Biol..

[34] Spina F., Volponi S. (2008). Atlante della Migrazione degli Uccelli in Italia: II.

[35] Ientile R., Massa B., Sicilia A. (2008). Uccelli (Aves). Atlante della Biodiversità della Sicilia: Vertebrati terrestri.

[36] Iapichino C., Massa B. (1989). The Birds of Sicily.

[37] Lindström Å., Svensson S., Green M., Ottvall R. (2007). Distribution and population changes of two subspecies of Chiffchaff *Phylloscopus collybita* in Sweden. Ornis Svec..

[38] Procházka P., Van Wilgenburg S.L., Neto J.M., Yosef R., Hobson K.A. (2013). Using stable hydrogen isotopes (δ2H) and ring recoveries to trace natal origins in a Eurasian passerine with a migratory divide. J. Avian Biol..

[39] Demongin L. (2016). Identification Guide to Birds in the Hand.

[40] Zduniak P., Yosef R., Bensusan K.J., Perez C.E., Tryjanowski P. (2015). Biometry and phenology of two sibling *Phylloscopus* warblers on their circum-Mediterranean migrations. ZooKeys.

[41] Doniol Valcroze P. (2024). On the taxonomic status of the Siberian Chiffchaff *Phylloscopus* [*collybita*] *tristis* (Phylloscopidae). Zootaxa.

[42] Ientile R., Tagliavia M., Cuti N., Termine R., Giannella C., Nissardi S., Zucca C., Cavaliere V., Lo Verde G., Campobello D. (2023). Morphometric versus genetic variation in the Eurasian Reed Warbler *Acrocephalus scirpaceus* in Italy. Bird Study.

[43] Zuccon D., Pons J.M., Boano G., Chiozzi G., Gamauf A., Mengoni C., Nespoli D., Olioso G., Pavia M., Pellegrino I. (2020). Type specimens matter: New insights on the systematics, taxonomy and nomenclature of the subalpine warbler (*Sylvia cantillans*) complex. Zool. J. Linn. Soc..

[44] Fraser D.J., Bernatchez L. (2001). Adaptive evolutionary conservation: Towards a unified concept for defining conservation units. Mol. Ecol..

